# Giant bleeding post-traumatic thoracic sarcoma management: A case report

**DOI:** 10.3389/fsurg.2022.1044077

**Published:** 2022-12-08

**Authors:** Alexey V Shabunin, Ivan N Lebedinsky, David D Dolidze, Zurab A Bagatelia, Serghei Covantsev, Dmitry S Bocharnikov, Nodar N Gogitidze

**Affiliations:** ^1^ Department of Surgery, Botkin Hospital, Moscow, Russia; ^2^ Department of General Surgery, Russian Medical Academy for Continuous Professional Education, Moscow, Russia

**Keywords:** thoracic soft tissue sarcomas, breast mobilization, bleeding, thorax, tumor

## Abstract

The heterogeneity of thoracic wall tumors often represents challenging clinical entities for surgeons due to diagnostic and treatment complexities. The primary tumors, metastases, or direct invasion from intrathoracic structures comprise almost half of all cases on average that are proved to be malignant. Surgery treatment usually leaves large chest defects that require further extensive reconstruction and multimodal management including radiotherapy and chemotherapy. We report a rare case of a giant (30 cm) post-traumatic bleeding thoracic sarcoma treatment in a 70-year-old female. The use of our modified Verneuil technique to close the extensive postoperative skin defect optimized surgical wound management and provided good functional and aesthetic results. Four-year follow-up outcomes after surgical and adjuvant radiation therapy reported a high level of tumor control and showed no evidence of postoperative disease recurrence.

## Introduction

Thoracic wall tumors are a rare heterogeneous group of neoplasms accounting for only 5% of all tumors in the thoracic anatomical area (1). They can be primary tumors, metastases, or direct invasion from intrathoracic structures and 50% of these neoplasms are malignancies (1). Thoracic soft tissue sarcomas are usually painless, slow-growing, and account for only 0.1%–0.15% of all chest malignancies (2).

These tumors are rather challenging for physicians because the primary treatment option such as surgery poses a significant and growing risk of morbidity and mortality (1). The standard-of-care treatment for thoracic wall tumors involves radiotherapy and chemotherapy. Emergency surgical treatment is not usually performed in such cases (3).

Another challenge that surgeons face is removal of giant bleeding tumors. The surgery usually leaves large chest defects that require extensive reconstruction and a multidisciplinary approach (4).

The largest soft-tissue sarcoma of the chest wall described by Davis et al. in 2016 was 27 cm (4). We present a rare case of a larger (30 cm) bleeding thoracic sarcoma management following antecedent traumatic injury.

## Case presentation

**Figure 1:**
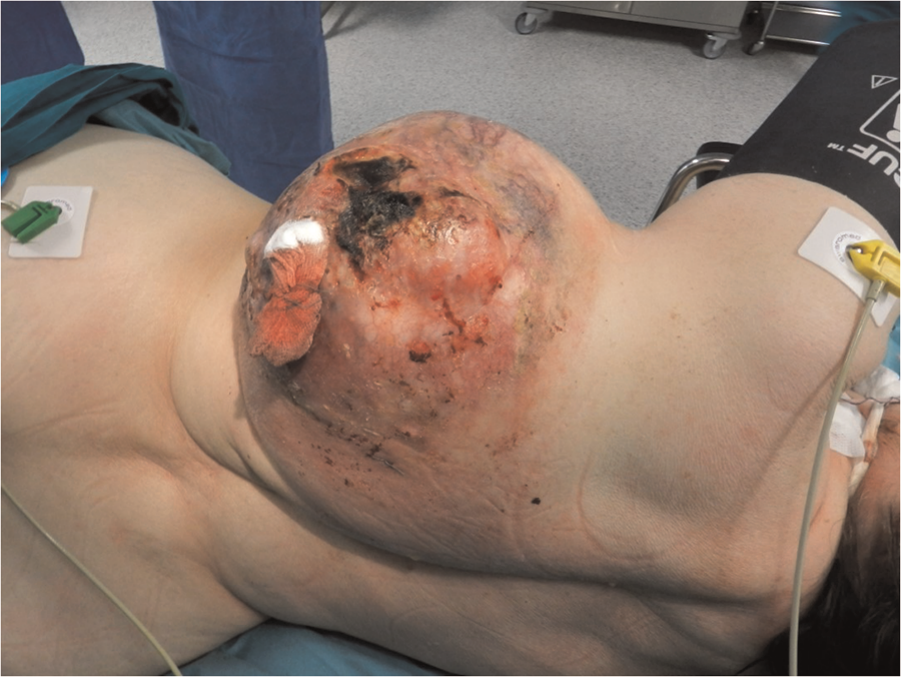
The bleeding necrotic tumor mass on the left posterolateral surface of the hemithorax.

A 70-year-old female was admitted to the hospital through the emergency department on January 17, 2018, with a giant intensely bleeding thoracic tumor after falling down the stairs and injuring the primary tumor site. According to the patient, the chest wall tumor showed slow growth for 20 years and enlarged significantly at the site of a traumatic injury after the incident. Some years ago, the patient refused surgical treatment plan as her previous healthcare provider had offered her a two-stage surgery with a long recovery period. On admission, her blood pressure was 140/70 mmHg, and her heart rate was 120 bpm. Physical examination revealed a bleeding necrotic tumor mass on the left posterolateral surface of the chest up to 30 cm in diameter and a necrotic wound approximately 10 cm in diameter. The skin immediately surrounding the tumor was hyperemic, moderately edematous, and indurated (Figure 1). The patient had a concomitant lower-extremity varicose vein disease - C1 score: telangiectasia according to the CEAP Classification (spider veins). Laboratory workup demonstrated anemia (erythrocytes 2.2 × 10^12^, [normal range 3.8–5.2 × 10^12^] hemoglobin 55 g/L [normal range 120–150 g/L], hematocrit 17.4% [normal range 35%–45%]), hypocoagulation (INR 1.3, normal range 0.8–1.1), and elevated liver transaminases levels (alanine transami nase 95.5 U/L [normal range 0–50 U/L], aspartate aminotransferase 91.1 U/L [normal range 0–50 U/L]). Electrocardiography showed sinus tachycardia and left bundle branch block. Other medical examinations were unremarkable. The tumor hemorrhage was controlled by stitching, cottonoids, hemostatic therapy (aminocaproic acid injection, 4000 mg intravenously), blood (2 units of 300 ml) and fresh frozen plasma (2 units of 300 ml) transfusions.

After clinical stabilization, we performed a thoracic and abdominal computer tomography (CT) scan with intravenous contrast media to assess the extent of the tumor. CT demonstrated a giant soft-tissue mass located in the left hemithorax at the T3-L1 vertebra. However, the projection of the dataset acquired on the CT scanner was truncated because the detected mass size extended outside the scan field of view.

**Figure 2:**
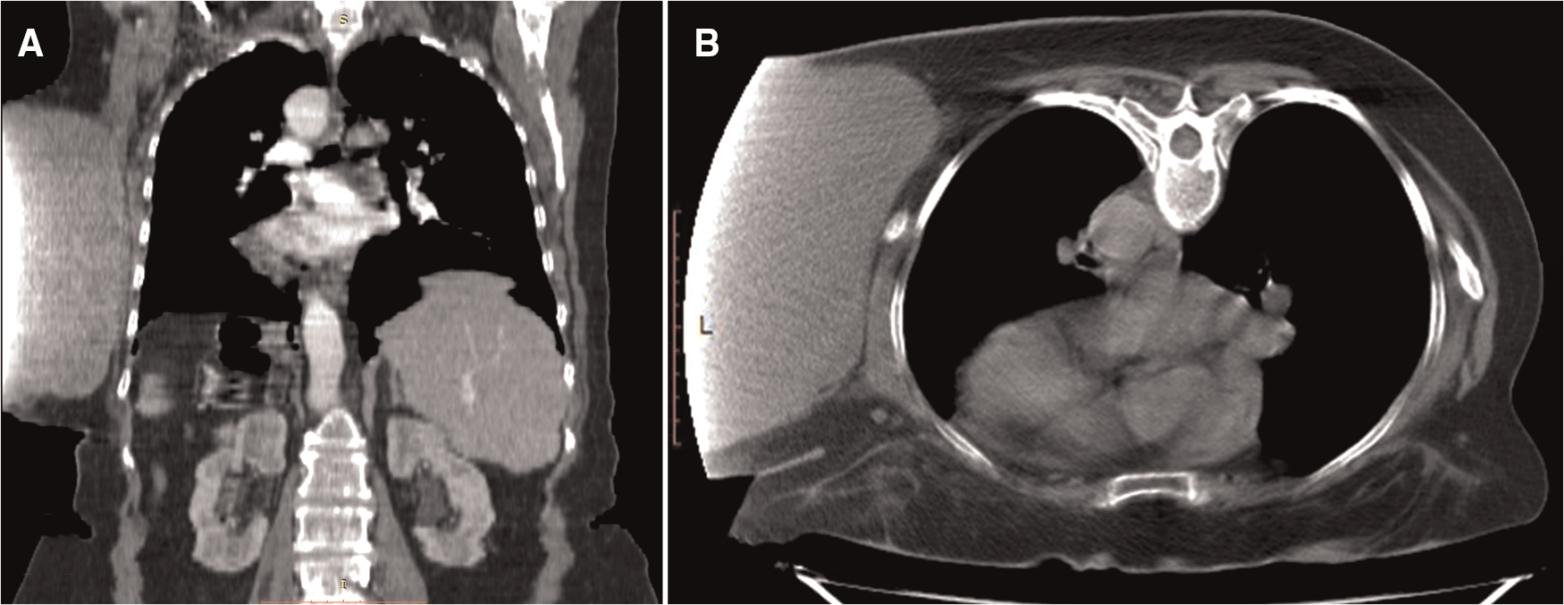
Chest soft tissue mass CT imaging. (**А**). Frontal view. (**B**). Axial view.

The tumor measuring 192 mm long and up to 178 mm wide was well-circumscribed, vascularized by dilated and tortuous vessels (Figure 2AB) and lay adjacent to the left costal surface of scapula and ribs without invading them. The left-sided axillary lymph nodes were not enlarged, and no bone destruction was detected. Additionally, the patient was also diagnosed with diffuse pneumosclerosis, atherosclerosis of the aorta and coronary arteries, and bilateral renal cysts (Bosniak I).

**Figure 3:**
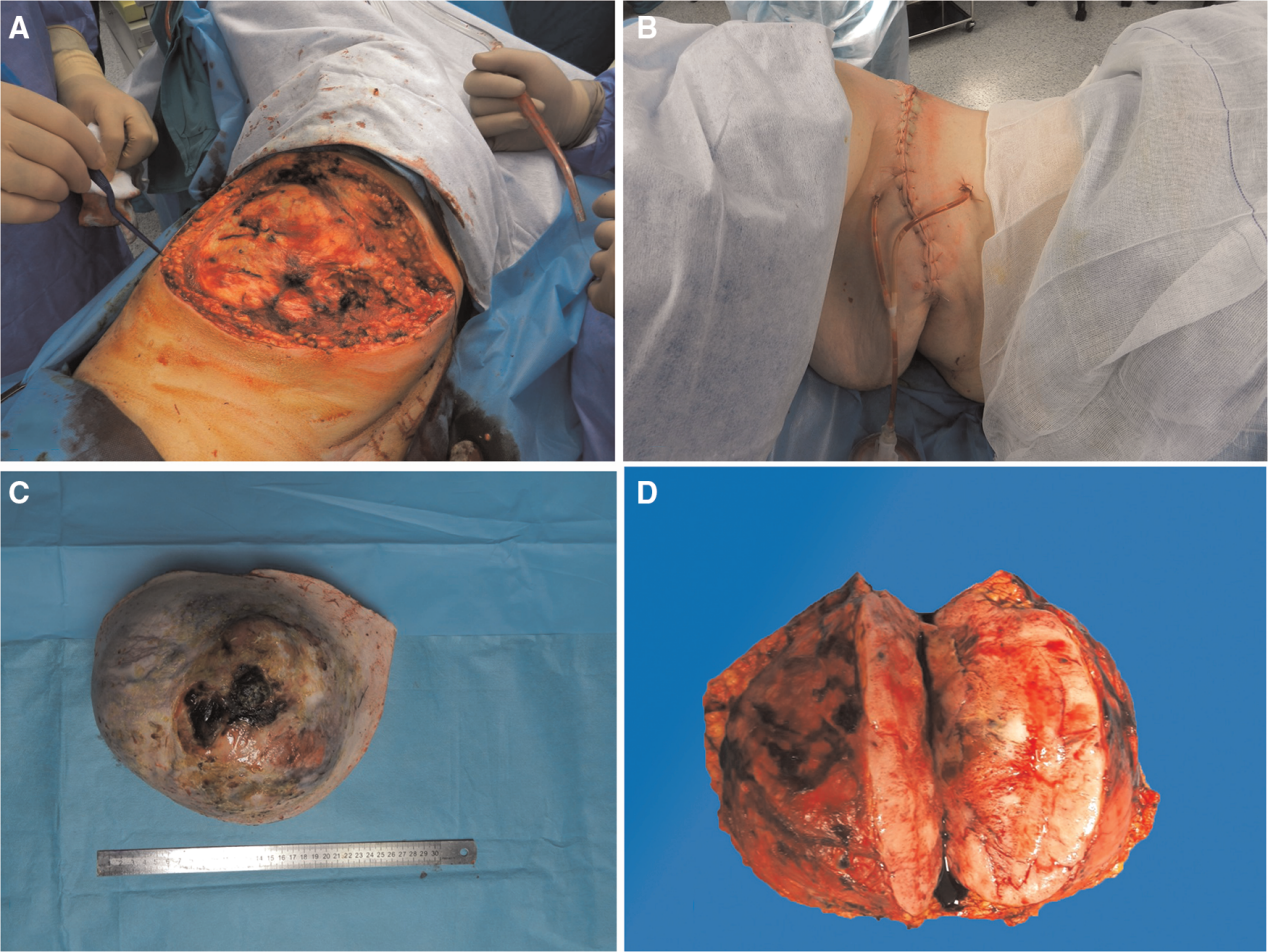
Intraoperative and postoperative findings. (**А**). Skin and tissue defects after tumor removal. (**В**). Postoperative view. Gross specimen of the mass. (**C**). Removed tumor. (**D**). The tumor cross section.

Preoperative assessment excluded the possibility of the chest wall invasion. On January 18 after clinical stabilization the tumor was removed with clear surgical margins (Figure 3A,B). According to the intraoperative evaluation, serratus inferior posterior muscles and the latissimus dorsi muscle were invaded by the tumor and required partial resection. The postoperative extensive skin and soft tissue defects were covered by the breast tissue mobilization toward the ipsilateral side (a modified Verneuil technique).

The surgically resected specimen showed a solid ulcerous-necrotic mass (Figure 3C,D). Histological analysis showed that a nodule of the spindle cell mesenchymal tumor structure, which was removed with clear surgical margins, had no signs of invasion into the muscle (R0). Immunohistochemistry (IHC) was performed for differential diagnosis of the spindle cell sarcomas group. IHC disclosed diffuse vimentin and positive CD34. S-100 protein, smooth muscle actin (SMA), and *β*-catenin markers were negative. Over 5 mitoses per field of view were detected.

Thus, according to morphologic findings the resected tumor was graded as an adult type fibrosarcoma ICD 8810/3. Considering the CD34 expression and the tumor localization, the solitary fibrous tumor had a risk of malignant transformation into a fibrosarcoma.

**Figure 4:**
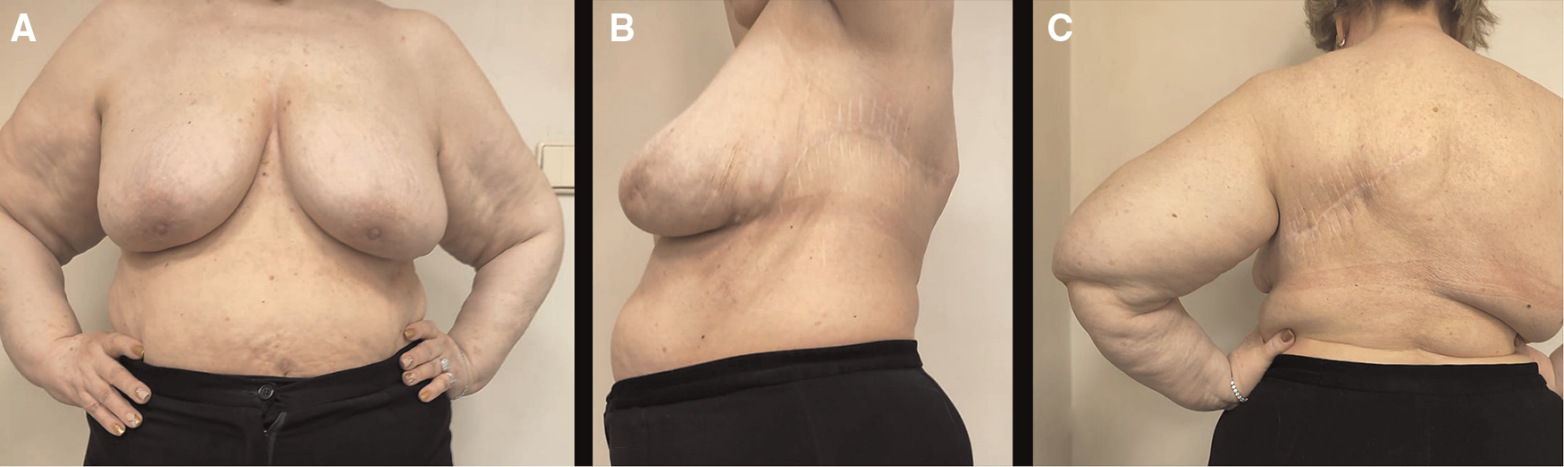
Four-year follow-up outcomes after surgical and radiation therapy. (**A**). Front view (Mammary glands are positioned at the same level). (**B**). Lateral view. (**С**). Back view.

The patient received conservative therapy postoperatively. The drainage tube was removed on day three after surgery. Postoperative ultrasonography of the soft tissues at the site of the surgical incision showed edema with single insignificant fluid segments up to 3–4 mm thick. A negative cumulative fluid balance achieved during hospitalization showed favorable clinical outcomes of the surgery and enhanced the patient's recovery. The postoperative laboratory test results improved significantly (erythrocytes: 3.49 × 10^12^, hemoglobin: 106 g/L, hematocrit: 30.4%). The patient was discharged in a satisfactory condition and assigned short-course radiotherapy for a total dose of 50 Gy. After 4 years of follow-up, no evidence of recurrence was observed (Figure 4A–C).

## Discussion

Thoracic wall tumors are usually rare and pose a challenge for the surgical team. Complexities of the diagnostic process, insufficient resection depth, inadequate and ineffective reconstruction of large chest wall defects can result in high perioperative mortality and morbidity (5).

Surgery is an essential part of the multimodal treatment scheme for thoracic sarcomas. However, the evidence of postoperative recurrence can be found in 23% of patients. Local recurrences occur in 8.7% of cases and the development of distant metastases (predominantly in the lungs) are diagnosed in 14.3% of cases (6). The 5-year relative survival rate for thoracic sarcomas is approximately 55%–66% (7, 8). The main factors that influence survival showed that tumor size <10 cm [odds ratio (OR) 3.95, *p* = 0.047], histologic grade 2 (OR 8.12, *p* = 0.004), and American Society of Anesthesiologists score 1 (OR 11.25, *p* = 0.001) are identified as independent predictors of actual 5-year survival (9). Complete tumor removal is one of the most important factors that can contribute to approximately 80% cases of survival (2). The mass size and the involvement of surrounding structures are the major challenges. In fact, the use of chemotherapy and radiotherapy have been shown largely futile in combating large soft-tissue sarcomas. Moreover, long-term damaging effects of radio- and chemotherapy may include ulceration, necrosis, and bleeding (3, 4). Therefore, radiotherapy is used to prevent tumor recurrence or to improve the treated result after radical resection that was not achieved (10).

Determining the preferred technique for large skin defects closure that follow the resection of huge neoplasms is paramount. Chest wall reconstruction is one of the most challenging procedures of the surgery process. It is indicated in a variety of clinical situations, such as rib resection (3 or more ribs), a postoperative soft-tissue defect measured more than 5 cm, the need for adequate chest wall stabilization and prevention of fluid accumulation and fistula formation, airtight closure (prevention of air leakage from the pleural space) and cosmetic appearance improvement. Muscle and musculocutaneous flaps are classically the tissues of choice to cover the wound (11, 12). Traditional techniques for wall chest reconstruction most commonly utilize vascularized free latissimus dorsi or pectoralis major flaps (13).

In the presented case the patient underwent vascularized free tissue transfer for thoracic chest wall reconstruction. To provide the most adequate result we used the breast soft tissues transfer as the most appropriate reconstruction method. There are several important factors that contributed to the choice of this option as a good alternative. Since the postoperative defect area size was more than 30 cm in diameter, a substantial amount of robust well-vascularized tissue for reconstruction was required to prevent postoperative necrosis of the flap. The vascularization of breasts is abundant and characterized by individual diversity. The main arteries that supply the gland are the internal thoracic artery, the lateral thoracic artery, the thoracoacromial artery, intercostal arteries and some of the vessels that supply nearby muscles (14). The presence of such an abundant arterial network makes it possible to use the breast tissue as a flap to close large tissue defects with lower risks for tissue necrosis due to tension of impaired vascular supply.

Breast mobilization is a surgical technique that implies the use of contralateral mammary gland to close large mastectomy defects (15). The original technique was introduced in 1,887 by Verneuil who used a breast pedicle flap for reconstruction of the opposite breast (16). A modification of this technique by the breast mobilization and transfer on the ipsilateral side made it possible to close an extensive skin and soft tissue defect in the left hemithorax.

The postoperative period was uneventful, and the postsurgical scar had no adverse outcomes. The tumor was completely removed, and the patient had no need for additional surgery or reconstructive procedures. Follow-up examinations within four years found no evidence of recurrence.

## Conclusions

The experience of the giant bleeding thoracic wall chest tumor treatment has shown that the most favorable outcome that can be achieved after surgery is a complete tumor resection in combination with a modified reconstructive surgery method which encompasses mobilization and tissue transfer of the breast towards the ipsilateral side.

The closure of skin defects after removal of a large bleeding wall chest tumor using the modification of Verneuil technique optimized postoperative surgical wound and incision healing, provided good functional and aesthetic results, and, therefore, improved the patient's quality of life.

## Data Availability

The original contributions presented in the study are included in the article/Supplementary Material, further inquiries can be directed to the corresponding author/s.
